# Perturbing neural stem cell fate in glioblastoma heterogeneity and beyond

**DOI:** 10.1016/j.stemcr.2025.102633

**Published:** 2025-09-09

**Authors:** Ethan W. Hollingsworth, Jaime Imitola

**Affiliations:** 1 Medical Scientist Training Program, Irvine, CA 92697, USA; 2 Department of Developmental and Cell Biology, University of California, Irvine, Irvine, CA 92697, USA; 3 Laboratory of Neural Stem Cells and Functional Neurogenetics, Farmington, CT 06030, USA; 4 Departments of Neuroscience, Neurology, Genetics and Genome Sciences, UConn Health, Farmington, CT 06030, USA

## Abstract

Intratumoral heterogeneity in glioblastoma is thought to underlie its remarkable ability to recur and resist therapies. Its origins, however, remain unknown. In this issue, Liu et al. model the contributions of cell-of-origin and genetic drivers to intratumoral heterogeneity in glioblastoma, using a perturbation paradigm with broad neurodevelopmental applications.

## Main text

Gliomas are glial cell-derived tumors affecting the brain and spinal cord that markedly range in their severity. The most severe are glioblastoma multiforme (GBM), a highly malignant and infiltrative class, exemplified by their poor prognosis and dismal survival rate (Weller et al., 2024). Current standard therapies include chemotherapy, radiation, and novel resection techniques, all with just modest efficacy. Despite the recent advances in our understanding of basic GBM biology, its high mortality and morbidity remain unchanged (Weller et al., 2024). Translating this knowledge into improved outcomes requires identifying the true drivers of GBM’s poor prognosis, including its remarkable ability to spread across the brain, resist therapies, and recur.

Once classified into four rigid molecular subtypes (pro-neural, neural, classical, and mesenchymal) (Verhaak et al., 2010), single-cell studies have revealed that all of these molecular subtypes co-exist within a single tumor simultaneously (Neftel et al., 2019). Instead, what differs among patients is the relative cellular proportions of each subtype, likely reflecting clonal, mutational, and patient-specific selective pressures. In other words, major cellular heterogeneity exists within and among these brain tumors.

Intratumoral heterogeneity is a leading hypothesis for the remarkable therapeutic resistance and recurrence of GBM, as treatments may effectively target one subtype but leave the others unscathed (Wang et al., 2022). The mechanisms underlying this heterogeneity, however, remain largely unknown. This is due to our lack of experimental access into a glioma’s evolution. Our window into tumor biology in humans only opens when patients present with clinical deficits or imaging abnormalities. By this time, proliferation has run wild, a tumor microenvironment has long been established, and we are left to try to retrace the tumor’s evolution and heterogeneity retrospectively using a single snapshot of data from a resected tumor specimen.

Rather than this retrospective approach, in this issue of *Stem Cell Reports*, Liu et al. (2025) sought to characterize intratumoral heterogeneity of brain tumors in the forward direction. The authors leveraged two key recent discoveries of glioma biology. First, the cells of origin vary by the type of glioma (i.e., neural stem cells [NSCs] underlie GBM) (Lee et al., 2018). Second, different oncogenic mutations are linked to different molecular subtypes (Neftel et al., 2019). These findings enabled them to ask how different cells of origin and various genetic drivers affect the intratumoral heterogeneity of gliomas

Focusing first on cells of origin, the authors used their recently developed sorting method to isolate NSCs, bipotent glial progenitors, and oligodendrocyte precursor cells (OPCs) from primary human fetal brain tissue (Liu et al., 2023). These isolated cells served as the starting point for their model before introducing two genetic perturbations typical of glioma, a dominant-negative TP53 and a knockdown of the tumor suppressor gene *NF1* (Reilly et al., 2000). Each of the progenitor cell types was then separately injected into a single hemisphere of immunodeficient mice to experimentally model how the cellular composition of these brain tumors evolves.

Through imaging and single-cell transcriptomics, the authors identified that these different cells of origin gave rise to tumors with varying cellular makeups. Mutant NSCs led to an increased proportion of neuron-like tumor cells while OPCs gave rise to predominantly oligodendroglial-like cells, including mature oligodendrocyte-like cells at later stages. These findings suggest that although p53 and *NF1* perturbations leave their proliferation unchecked, each progenitor largely maintains its typical cell fate.

**Figure 1:**
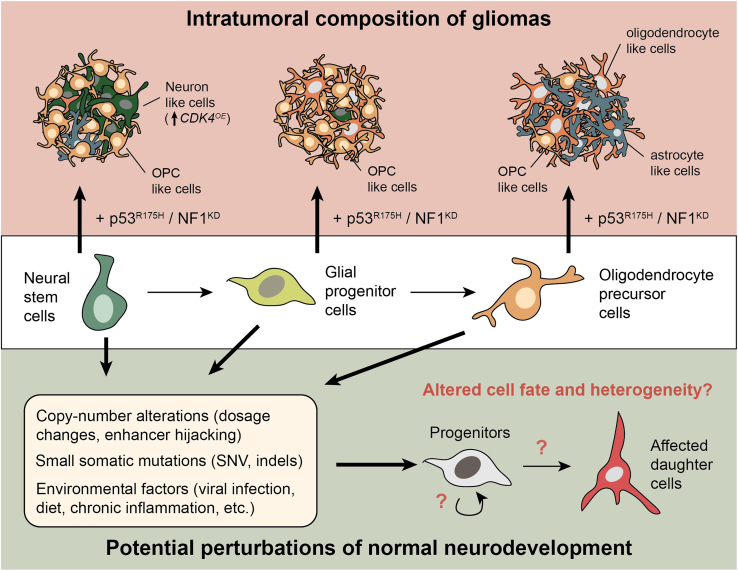
Dissecting cell and genetic drivers of heterogeneity in cell fate choices in glioma and development

Disrupting just the cell cycle might not be enough to skew the fates of these progenitors away from their normal lineages. This led the authors to test how the overexpression of genes linked to specific subtypes of GBM would affect intratumoral heterogeneity of mutant NSCs. Strikingly, overexpressing *CDK4* biased 88% of p53-mutant NSCs toward a neuron-like subtype, consistent with its past link (Neftel et al., 2019). The effects of overexpressing *EGFR* (linked to the astrocytic subtype) and *PDGFRA* (linked to the OPC subtype) on intratumoral heterogeneity were less drastic and did not result in as much of a subtype bias as seen by the authors in GBM *in vivo*. The underlying reason for this discrepancy is unclear but could be due to a lack of immune involvement in these nude mice, a human-specific extrinsic mechanism, insufficient engraftment time, or some other unknown factor. Nevertheless, their results robustly highlight the individual contributions of cell-of-origin and genetic drivers on intratumoral heterogeneity (Figure 1).

The work performed here—combining primary cell isolation techniques with clinically relevant *in vivo* models of gliomagenesis—lays the technical foundation for delving into mechanistic questions into how a single tumor progenitor gives rise to a heterogeneous collection of daughter cells. In the context of gliomas, the authors demonstrate that such capacity is largely encoded at the level of the cell type, consistent with findings from patient-derived xenografts, which likewise show a sustained tendency toward specific molecular subtypes (Xie et al., 2024b). Though only testing three oncogenetic drivers, only *CDK4* drastically changed the fate decisions of mutant NSCs while *EGFR* and *PDGFRA* showed lesser effects. This raises the critical question of what intrinsic and extrinsic factors influence lineage choice of GBM progenitors? Intrinsically, it could be that large-scale structural changes are required to sufficiently induce new regulatory programs via enhancer hijacking (Xie et al., 2024a). Extrinsically, clonal biases in heterogeneity may arise from urgent selective pressures induced by chemotherapies and radiation (Wang et al., 2022). Identifying the molecular underpinnings by which tumor cells adapt to such pressures will be critical. Combining the author’s current model with single-cell and long-read genomics sequencing, lineage tracing-based techniques, and modeling the effects of standard chemotherapies should start to uncover some of these mechanisms.

From a broader perspective, the ideas tested here may also be helpful for thinking of how heterogeneity emerges or goes awry during normal development. The authors’ overexpression paradigm, for instance, mimics testing the effects of germline copy-number variation on fate choice. The importance of dosage in this process is underscored by many haplo- and triplo-sensitive human genes encoding lineage-specifying transcription factors, which, when perturbed, disrupt cell fate (Collins et al., 2022). Somatic, but non-cancerous, mutations in NSCs, on the other hand, may also skew cell fate decisions toward a particular lineage. Likewise, exposure to environmental perturbations like viruses or chronic neuroinflammation may globally dysregulate normal NSC chromatin states, causing a bias toward certain trajectories. These skewed choices could disrupt the overall cellular architecture of the normal brain with damaging repercussions for circuit assembly during development or adult CNS repair. Borrowing this idea of perturbations in cancer, the stem cell field can try to see past stochasticity as a lone explanation for heterogeneity and strive to identify intrinsic and extrinsic genetic regulators of normal and diseased NSC and progenitor fate decisions.

## Declaration of interests

The authors declare no competing interests.
